# Drug-Related Lymphedema: Mysteries, Mechanisms, and Potential Therapies

**DOI:** 10.3389/fphar.2022.850586

**Published:** 2022-03-04

**Authors:** Soumiya Pal, Jenat Rahman, Shengyu Mu, Nancy J. Rusch, Amanda J. Stolarz

**Affiliations:** ^1^ Department of Pharmaceutical Sciences, College of Pharmacy, University of Arkansas for Medical Sciences, Little Rock, AR, United States; ^2^ Department of Pharmacology and Toxicology, College of Medicine, University of Arkansas for Medical Sciences, Little Rock, AR, United States

**Keywords:** lymphedema, cardiovascular drugs, potential therapeutic agents, chemotherapy drugs, antidiabetic drugs, lymphatic function

## Abstract

The lymphatic circulation is an important component of the circulatory system in humans, playing a critical role in the transport of lymph fluid containing proteins, white blood cells, and lipids from the interstitial space to the central venous circulation. The efficient transport of lymph fluid critically relies on the rhythmic contractions of collecting lymph vessels, which function to “pump” fluid in the distal to proximal direction through the lymphatic circulation with backflow prevented by the presence of valves. When rhythmic contractions are disrupted or valves are incompetent, the loss of lymph flow results in fluid accumulation in the interstitial space and the development of lymphedema. There is growing recognition that many pharmacological agents modify the activity of ion channels and other protein structures in lymph muscle cells to disrupt the cyclic contraction and relaxation of lymph vessels, thereby compromising lymph flow and predisposing to the development of lymphedema. The effects of different medications on lymph flow can be understood by appreciating the intricate intracellular calcium signaling that underlies the contraction and relaxation cycle of collecting lymph vessels. For example, voltage-sensitive calcium influx through long-lasting (“L-type”) calcium channels mediates the rise in cytosolic calcium concentration that triggers lymph vessel contraction. Accordingly, calcium channel antagonists that are mainstay cardiovascular medications, attenuate the cyclic influx of calcium through L-type calcium channels in lymph muscle cells, thereby disrupting rhythmic contractions and compromising lymph flow. Many other classes of medications also may contribute to the formation of lymphedema by impairing lymph flow as an off-target effect. The purpose of this review is to evaluate the evidence regarding potential mechanisms of drug-related lymphedema with an emphasis on common medications administered to treat cardiovascular diseases, metabolic disorders, and cancer. Additionally, although current pharmacological approaches used to alleviate lymphedema are largely ineffective, efforts are mounting to arrive at a deeper understanding of mechanisms that regulate lymph flow as a strategy to identify novel anti-lymphedema medications. Accordingly, this review also will provide information on studies that have explored possible anti-lymphedema therapeutics.

## 1 Introduction

Lymphedema is a disease that impacts approximately 10 million people in the United States and hundreds of millions of individuals worldwide (Lymphatic Education and Research Network, 2019). It is a chronic debilitating condition characterized by fluid accumulation in the interstitial spaces due to insufficient lymphatic drainage that ultimately leads to swelling, pain, and discomfort (Lymphedema Side Effects of Cancer Treatment, 2021 | CDC; PDQ Supportive and Palliative Care Editorial Board, 2002; Sleigh and Manna, 2021). Lymphedema can be classified as either *primary lymphedema* to describe a genetic condition caused by abnormalities in development of the lymphatic system, or *secondary lymphedema* referring to an acquired condition resulting from damage to the lymphatic system (Supportive and Palliative Care Editorial Board, 2002; Warren et al., 2007; Sleigh and Manna, 2021). According to epidemiological data, primary lymphedema affects one in 100,000 individuals under 20 years of age (Smeltzer et al., 1985; Rockson and Rivera, 2008), whereas secondary lymphedema is more common and one in 1,000 Americans may suffer from this condition (Rockson and Rivera, 2008; Sleigh and Manna, 2021). Any injury or insult that results in partial or full obstruction of lymph flow from the interstitial space to the venous circulation can cause secondary lymphedema. For example, *Wuchereria bancrofti* and *Brugia malayi* or *timori* mediated filariasis, which is a tropical disease in which the adult worms reside in the lymphatic circulation and block lymph flow, is the most common cause of secondary lymphedema globally (Ottesen, 1985; Tan et al., 1985; Baird et al., 1986). In contrast, the high prevalence of secondary lymphedema in developed countries is primarily caused by malignancies or chemotherapy medications (Rockson et al., 2019).

Collectively, many risk factors in addition to those mentioned above have been linked to the development of secondary lymphedema. Some of these factors and conditions include obesity, lymph node dissection, radiation therapy, lymph node biopsy, infection, peripheral vascular surgery, burn scar excision, vein stripping, and lipectomy (Slater et al., 2013; Ugur et al., 2013; Sleigh and Manna, 2021). Additionally, there is growing recognition that many pharmacological agents that bind to ion channels and other signaling molecules that mediate the cyclic contraction and relaxation of lymph muscle cells also may contribute to the development of lymphedema (Tesar and Armer, 2018). In some instances, preclinical studies have provided compelling evidence to implicate specific drugs as disrupters of cellular signaling pathways in lymph muscle cells required for rhythmic contractions and healthy lymph flow. Epidemiological studies designed to identify factors contributing to the development of drug-related lymphedema in patients often are less conclusive due to confounding comorbid conditions that also may disrupt fluid balance. However, the collective findings from these studies provide additional strong evidence that drug-related lymphedema is a prevalent condition with many common medications implicated as risk factors.

Unfortunately, there are no FDA -approved pharmacological treatments for secondary lymphedema; however, with timely diagnosis and treatment it can be managed to prevent or slow progression. In fact, the International Society of Lymphology has issued guidelines for the proper management of lymphedema to improve quality of life (Lymphology, 2020). This management primarily focuses on edema of the limbs, which is most visible and physically available for intervention. Depending on the severity and site of the lymphedema, management may include complete decongestive therapy with the help of manual lymphatic drainage and multilayer bandaging, exercises, compression garments, pneumatic compression pumps, low level laser therapy, far infrared radiation, negative pressure therapy, extracorporeal shock wave therapy, stellate ganglionic block, vascular lymph node transfer, lymphatico-venous anastomosis, lymphatico-lymphatic bypass, and suction-assisted protein lipectomy. Proper diet, self-care like proper skin care, and education about the disease also are essential for proper management (Borman, 2018). Interestingly, these guidelines do not mention drug therapies that potentially may contribute to the development and maintenance of lymphedema.

Diseases involving the lymphatic circulation have received scant attention in the past compared to pathologies involving the blood circulation that have been the focus of intense scrutiny. However, new advances in the field of lymphatic biology have begun to shed light on the diverse causes and pathophysiological mechanisms of secondary lymphedema. The purpose of this review is to discuss briefly some fundamental properties of lymph muscle cells that determine lymph flow, and then examine the evidence for drug-related lymphedema with an emphasis on common medications administered to treat cardiovascular diseases, metabolic disorders, and cancer. Understanding the mechanisms by which drugs contribute to the development of lymphedema will provide a rational basis for avoiding certain drug families when planning medication regimens for patients at risk for lymphedema. Additionally, although current pharmacological approaches used to prevent and/or reverse lymphedema have not proven very effective, we also will review studies that have evaluated diuretics, hyaluronidase, ion channel modulating agents, ketoprofen, and tacrolimus, as possible anti-lymphedema therapeutics.

## 2 Lymphatic Muscle Cell Physiology and Excitability

The effect of different medications on lymph flow can be understood by appreciating the intricate intracellular calcium signaling that underlies the contraction and relaxation cycle of collecting lymph vessels. The primary “mechanical” job of the lymphatic vasculature is to propel fluid (containing protein, lymphocytes, lipids, and other cells) from the extracellular space to the central venous ducts to prevent excess fluid accumulation in tissues (Schmid-Schonbein, 1990; Aukland and Reed, 1993; Gashev, 2002; Zawieja, 2009; Choi et al., 2012; Hansen et al., 2015; Scallan et al., 2016). The lymphatic vasculature begins with blind-ended lymph capillaries intertwined with the vascular capillary beds, which drain into collecting lymph vessels. The collecting lymph vessels are composed of a single inner layer of endothelial cells surrounded by a single layer of specialized lymphatic muscle cells (LMCs) (Gashev, 2002; Muthuchamy et al., 2003; Ji, 2008; Van Helden, 2014). Collecting lymphatics also contain bicuspid valves to prevent the back-flow of lymph fluid during contraction and the valves also serve to separate lymph vessels into functional units termed lymphangions (Davis et al., 2011; Van Helden, 2014). The transport of lymph fluid relies on contributions from intrinsic and extrinsic forces. The extrinsic forces relate primarily to factors that determine passive lymph transport and include the formation of new lymph fluid, arterial pulsations, and central venous pressure fluctuations. Passive lymph transport also is driven by site-specific forces. For example, transport through lymph vessels buried in skeletal muscle can be facilitated by muscle contraction, whereas respiration can drive lymph transport through lung lymphatics (Schmid-Schonbein, 1990; Negrini et al., 1994; Bridenbaugh et al., 2003).

Although extrinsic forces play a role in lymph transport *in vivo*, most studies regarding lymphatic function have focused on mechanisms of active lymph transport mediated by the intrinsic lymphatic pump. The intrinsic lymphatic pump underlies the spontaneous rhythmic contractions of collecting lymph vessels and is thought to rely on pacemaker muscle cells, which may compose 1–5% of the muscle cell population in the lymph vessel wall (Baron et al., 1991; Van Helden, 1993; Imtiaz et al., 2007). The remaining lymph muscle cells have been described as a specialized hybrid of smooth muscle and striated muscle since they express phenotypic properties and molecular markers of both muscle types (Muthuchamy et al., 2003; Dougherty et al., 2008; Breslin, 2014; Scallan et al., 2016).

The electrophysiological profile of lymph muscle cells underlies the physiological process of cyclic contraction and relaxation of collecting lymph vessels that drives lymph flow. Most of our knowledge is drawn from initial reports exploring the basis of spontaneous excitability in isolated lymph vessels. The resting membrane potential (E_m_) of muscle cells in the guinea pig mesenteric lymphatic circulation approximates −55 mV and spontaneous depolarizations of 30–40 mV occur under resting conditions (Van Helden, 1993; Imtiaz et al., 2007). Pharmacological evidence suggests that the level of resting E_m_ is established by potassium efflux through voltage-sensitive potassium channels (Allen et al., 1988; Telinius et al., 2014c) and chloride influx through calcium-activated chloride channels (Toland et al., 2000; Imtiaz et al., 2007). The cellular events that cause lymphatic pacemaker cells to spontaneously depolarize as the source for excitation and contraction of the lymph muscle syncytium are poorly understood. It’s been suggested that the binding of an endothelium-derived peptide (endothelin-1) to its plasma membrane receptor on lymph muscle cells may trigger calcium release from inositol trisphosphate receptors. The subsequent chloride influx through calcium-sensitive chloride channels is proposed to mediate membrane depolarization, which opens voltage-gated L-type calcium channels to provide the rise in cytosolic free calcium that initiates contraction (Azuma et al., 1977; Baron et al., 1991; Van Helden, 1993; Atchison and Johnston, 1997; Toland et al., 2000; Zhao and Van Helden, 2003; Ferrusi et al., 2004; Beckett et al., 2007; Imtiaz et al., 2007). Notably other types of ion channels similar to those that mediate pacemaker function in the sino-atrial node of the heart have been identified in lymph muscle cells, but the roles of these channels in lymphatic pacemaker activity are unclear. For example, sodium channels are proposed to contribute to action potential generation and contraction in lymph vessels (Hollywood M. A. et al., 1997; Telinius et al., 2014a, 2015), they will be discussed in more detail in later sections (see Section 3.1.1). Other ion channels that are expressed by lymph muscle cells include transient (T-type) calcium channels (Hollywood M. A. et al., 1997; Lee et al., 2014), hyperpolarization-activated cyclic nucleotide-gated (HCN) channels (McCloskey et al., 1999), and *ether-à-go-go* related gene (ERG) channels (Gui et al., 2014).

Although the precise cellular events that mediate the pacemaker current in lymph muscle cells are unclear, it is well documented that spontaneous depolarization of lymph muscle cells opens voltage-sensitive L-type calcium channels in the plasma membrane to cause Ca^2+^ influx, activation of contractile proteins, and ultimately the rhythmic contractions of lymph vessels (Azuma et al., 1977; Schmid-Schonbein, 1990; Van Helden, 1993; Gashev, 2002; Zawieja, 2009; Nipper and Dixon, 2011; Choi et al., 2012). Accordingly, pharmacological antagonists of the L-type calcium channel that include clinically-used “calcium channel blockers” effectively inhibit lymphatic contractions (Imtiaz et al., 2007; Von Der Weid et al., 2008; Telinius et al., 2014c; Lee et al., 2014). The transient rise in intracellular calcium mediated by the opening of L-type calcium channels during spontaneous depolarization of lymph muscle cells also may open inositol trisphosphate receptors and ryanodine receptors to mobilize intracellular calcium stores (Atchison and Johnston, 1997; Atchison et al., 1998; Zhao and Van Helden, 2003; Imtiaz et al., 2007; Jo et al., 2019; Stolarz et al., 2019). Ultimately, potassium efflux restores resting membrane potential and facilitates relaxation of lymph muscle cells (Cotton et al., 1997; Telinius et al., 2014b). A number of studies have reported roles for different potassium channels in regulating the rhythmic contractions of isolated lymph vessels or thoracic ducts from rats, guinea pig and humans (Mizuno et al., 1999; Mathias and von der Weid, 2013; Telinius et al., 2014b). For example, non-selective pharmacological block of potassium channels had no effect on contractions in isolated rat mesenteric lymph vessels (Mizuno et al., 1999), but contraction amplitude and frequency was increased in isolated human thoracic ducts (Telinius et al., 2014b). Pharmacological block of large-conductance potassium channels also increased the amplitude and frequency of contractions in isolated human thoracic ducts (Telinius et al., 2014b) and similar responses were recorded after block of voltage-sensitive potassium channels (Telinius et al., 2014b). Finally, ATP- sensitive potassium (K_ATP_) channels also have been identified in lymph muscle cells. Glibenclamide -induced block of these channels reportedly depolarizes guinea pig lymph muscle cells but has little to no effect on contractions of isolated rat mesenteric lymph vessels and isolated human thoracic ducts (Mizuno et al., 1999; Mathias and von der Weid, 2013; Telinius et al., 2014b). Although K_ATP_ channels may be inactive under some conditions, they can cause hyperpolarization, inhibition of rhythmic contractions, and loss of lymph flow when activated by pharmacological agents or gain-of-function mutations (Mathias and von der Weid, 2013; Davis et al., 2020; Garner et al., 2021).

**FIGURE 1:**
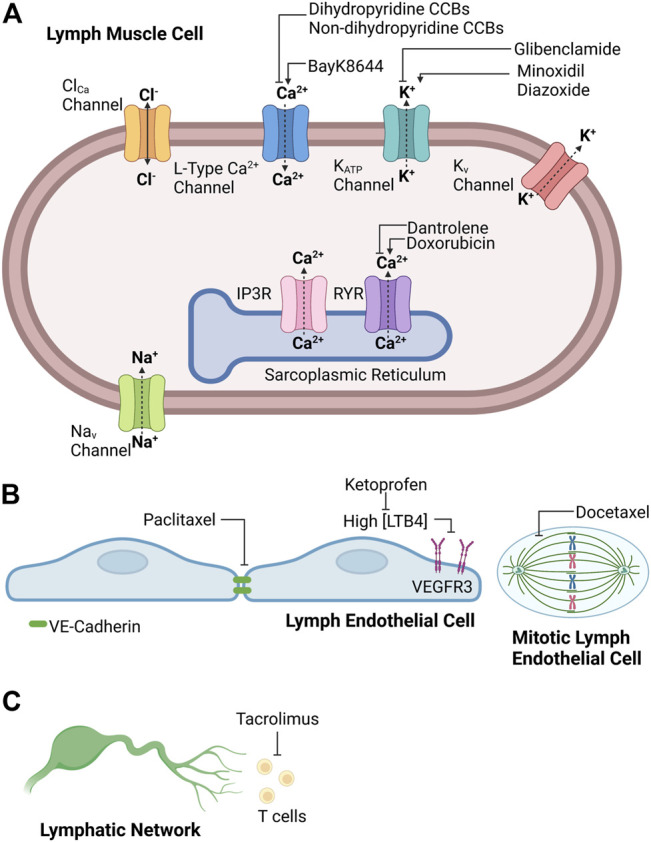
Potential sites of drug action in **(A)** lymph muscle cells, **(B)** lymph endothelial cells, and **(C)** lymphatic network. Created with BioRender.com.

The strict cyclic regulation of membrane potential and intracellular calcium signaling in lymph muscle cells ensures the presence of robust rhythmic contractions and relaxations, which ultimately provide the intrinsic driving force for lymph flow. For this reason, pharmacological agents that disrupt the events that underlie effective intrinsic “pumping” of lymph fluid will reduce lymph flow and predispose to the development of lymphedema. It is recognized that some clinically used medications with cellular targets implicated in lymphatic contractions may contribute to the development of lymphedema. However, the precise incidence of drug-related lymphedema caused by these agents still is being investigated and individual agents will be discussed in more detail in later sections. Interestingly, the frequency of drug-related peripheral edema, which may include lymphedema, associated with these medications may range from 3 to 64% depending on agent, dose, and duration (Keeley, 2008). The following sections of this review will examine literature implicating different classes of medications in the pathogenesis of secondary lymphedema and discuss efforts to identify an effective treatment to prevent or alleviate this debilitating condition. Accordingly, Figure 1 provides a schematic summary for potential sites of drug action in the lymphatic circulation, and Table 1 summarizes the effect of the common clinically used drugs on the lymphatic circulation that are included in this review.

**TABLE 1:** Effect of common clinical drugs on the lymphatic circulation.

Drug class	Agents	Species	Lymphatic bed	Effect	References
Sodium Channel Blockers	Ranolazine	Human	Mesenteric lymph vessels	No effect on contractions *ex vivo*	Telinius et al. (2015)
L-type Calcium channel Blockers (CCBs)		Human breast cancer patients		Increased risk of lymphedema	Stolarz et al. (2019a)
		Human		Formation of peripheral edema	Makani et al. (2011)
CCB- Dihydropyridine	Amlodipine	Human (female)	Lower limb lymph vessels	Participants with reduced baseline lymph flow *in vivo* developed lower limb edema	Mohanakumar et al. (2020)
	Nifedipine	Mice	Inguinal axillary and popliteal lymphatics	Reduced contraction amplitude, slightly increased frequency *ex vivo*	To et al. (2020)
	Nifedipine	Guinea pig	Mesenteric lymph vessels	Inhibited action potentials *ex vivo*	Imtiaz et al. (2007)
	Nifedipine	Guinea pig	Mesenteric lymph vessels	Inhibited action potentials *ex vivo*	Von Der Weid et al. (2008)
	Nifedipine	Guinea pig	Mesenteric lymph vessels	Inhibited contractions *ex vivo*	Zhao and Van Helden, (2003)
	Nifedipine	Rat	Mesenteric lymph vessels	Reduced force of contractions *ex vivo*	Lee et al. (2014)
	Nifedipine	Cow	Mesenteric lymph vessels	Inhibited contractions *ex vivo*	Atchison and Johnston, (1997)
	Nifedipine	Human	Thoracic ducts	Inhibited contractions *ex vivo*	Telinius et al. (2014c)
			Mesenteric lymph vessels	No reduction in lymph flow *in vivo*	
			Lower limb lymph vessels		
	Diltiazem	Rat	Mesenteric lymph vessels	Reduced force of contractions *ex vivo*	Lee et al. (2014)
	Diltiazem	Cow	Mesenteric lymph vessels	Inhibited contractions *ex vivo*	Atchison and Johnston, (1997)
	Verapamil	Human	Thoracic ducts	Inhibited contractions *in vitro*	Telinius et al. (2014c)
			Mesenteric lymph vessels		
K_ATP_ channel openers	Diazoxide	Rat	Mesenteric lymph vessels	Inhibited contractions *ex vivo*	Garner et al. (2021)
	Minoxidil sulfate	Rat	Mesenteric lymph vessels	Inhibited contractions *ex vivo*	Garner et al. (2021)
				Reduced lymph flow *in vivo*	
Thiazolidinedione	Rosiglitazone	Mice	Tumor lymph vessels	Tumor lymph vessel density was reduced *in vivo*	Chen et al. (2013)
	Rosiglitazone	Rats	Hind limb lymph vessels	Modest decreased lymphatic uptake *in vivo*	Cosson et al. (2009)
Anthracyclines		Human		Increased incidence of lymphedema in breast cancer patients	Norman et al. (2010)
					Ahmed et al. (2011)
					Bevilacqua et al. (2012)
					Nguyen et al. (2017)
	Doxorubicin	Rat	Mesenteric lymph vessels	Inhibited *ex vivo* contractions	Stolarz et al. (2019)
				Disrupted Ca^2+^ signaling *ex vivo* and reduced lymph flow *in vivo*	Van et al. (2021)
Taxanes		Human		Increased incidence of lymphedema in breast cancer patients	Lee et al. (2014)
					Nguyen et al. (2017)
	Docetaxel	Human	Dermal Lymphatic Endothelial Cells	Decreased HDLEC proliferation, and inhibited HDLEC migration and tubule formation, and decreased LYVE-1 expression *in vitro*	Wong et al. (2020)
	Paclitaxel	Mice	Tumor lymph vessels	Decrease in tumor lymphangiogenesis	Zamora et al. (2019)
	Paclitaxel	Human	Dermal Lymphatic Endothelial Cells	Induced autophagy, increased intracellular gaps, and loss of VE-cadherin *in vitro*	Zamora et al. (2019)

## 3 Cardiovascular Agents

Different classes of cardiovascular agents may exert off-target effects on the lymphatic circulation due to the presence of signaling pathways shared by the targeted tissue and the lymphatic muscle cells. Indeed, a descriptive cross-sectional study comparing self-reported co-morbid conditions and medication use between female breast cancer patients with and without arm lymphedema reported that the use of medications to treat cardiovascular diseases including cardiac arrhythmias, hypertension and heart failure were associated with an increased risk of lymphedema (Ridner and Dietrich, 2008). However, the medications in this study only were classified into broad therapeutic categories of cardiovascular agents rather than delineated by drug class or mechanistic action, which has confounded efforts to correlate specific drug-elicited signaling pathways to lymphedema risk. Here, we review the available evidence implicating specific classes of cardiovascular drugs in the development of lymphedema with an emphasis on vertically integrating drug mechanism of action with impact on lymphatic contractions and ultimately clinical evidence of interstitial fluid imbalance.

### 3.1 Anti-Arrhythmic Agents

Anti-arrhythmic agents are divided into four classes based on the mechanism by which they target distinct ion channels or receptors expressed by myocardial cells to restore normal rhythm and conduction in the heart. These four classes include sodium channel blockers (Class I), beta adrenergic receptor (βAR) blockers (Class II), potassium channel blockers (Class III), and calcium channel blockers (Class IV). Epidemiological studies suggest that administration calcium channel blockers (CCBs) increase the risk of lymphedema (Stolarz et al., 2019a), and additionally Class I and Class III antiarrhythmic drugs may have the potential to disrupt generation of the action potentials and rhythmic contractions in lymph muscle cells.

#### 3.1.1 Sodium Channel Blockers (Class I)

Although calcium influx through L-type calcium channels is the major contributor to the upstroke of the action potential and the amplitude of rhythmic contractions in isolated lymph vessels, antagonists of voltage-gated sodium channels also have been reported to attenuate contraction amplitude in isolated lymph vessels from several animal models (Smith, 1949; McHale et al., 1980; Ohhashi et al., 1980; Russell et al., 1980; Ohhashi and Roddie, 1981; Harty et al., 1993; Hollywood MA. et al., 1997; Igarashi et al., 1998). Similarly, sodium influx through voltage-gated sodium channels also is reported to contribute to the action potential and corresponding spontaneous contractions in isolated human thoracic ducts and mesenteric lymph vessels (Telinius et al., 2014a, 2015). Interestingly, rhythmic contractions were attenuated by the non-specific sodium channel blocker tetrodotoxin (TTX) in a subset of human mesenteric lymphatic vessels, whereas the clinically used sodium channel blocker ranolazine had no significant effect. This difference may be related to the predominant expression of the Na_v_1.3 isoform in human lymph vessels (Telinius et al., 2015), since ranolazine selectively blocks several sodium channel isoforms (Na_v_1.4, 1.5, 1.7, and 1.8) but fails to block Na_v_1.3 whereas TTX is a non-selective sodium channel antagonist that inhibits all isoforms. Accordingly, the clinical use of ranolazine and other sodium channel blockers have not been associated with lymphedema (Dokken and Fairley, 2021), although recent efforts to develop Na_v_1.3 selective blockers raise the possibility that these medications may have unintended effects on the lymphatic circulation (Keppel Hesselink, 2017; Pryde et al., 2017; Su et al., 2017).

#### 3.1.2 Beta Adrenergic Receptor Blockers (Class II)

The βAR blockers (β-blockers) are a class of widely used anti-arrhythmic and antihypertensive medications that are firmly linked to fluid retention. Treatment guidelines recommend against initiating β-blocker therapy in patients exhibiting signs of fluid overload due to possible exacerbation of this condition. Currently, it’s unclear whether the fluid retention relates to an effect of the β-blockers on the lymphatic circulation or instead can be fully attributed to effects on the heart and blood vasculature. In this regard, the peripheral edema associated with the administration of β-blockers has been attributed traditionally to the negative chronotropic and inotropic effects of these medications on the heart, which may increase venous blood volume and subsequently elevate capillary pressure to drive fluid into the interstitial space. Additionally, some β-blockers including labetalol, carvedilol, and nebivolol have direct vasodilator properties as an added mechanism of increasing capillary pressure. However, evidence suggests that lymph vessels also are innervated by sympathetic nerves and express βARs that may play a role in the rate and pattern of rhythmic contractions and lymph muscle tone (Thornbury et al., 1989; Ikomi et al., 1991). To date, no preclinical studies have directly evaluated changes in lymph flow *in vivo* in response to β-blocker therapy.

#### 3.1.3 Potassium Channel Blockers (Class III)

Potassium channels are densely expressed in cardiac myocytes and Class III anti-arrhythmic agents attenuate potassium efflux primarily by blocking voltage-gated potassium channels to prolong the repolarization phase of the cardiac action potential. As discussed earlier (see Section 2), potassium efflux mediated by at least several types of potassium channels also contributes to the resting membrane potential of lymph muscle cells and mediates repolarization of the action potential to ensure normal rhythmicity of spontaneous contractions. Importantly, pharmacological block of voltage-sensitive potassium channels, the main target of the Class III anti-arrhythmic drugs, is reported to elicited a biphasic response in lymph muscle cells; low antagonist concentrations increase contraction frequency without altering other contractile parameters, whereas higher antagonist concentrations transiently increase contraction frequency and progressively enhance contraction amplitude (Telinius et al., 2014b). Both of these effects caused by blocking voltage-sensitive potassium channels would be expected to increase rather than decrease lymph flow. In accordance, administration of Class III antiarrhythmic medications has not been associated with peripheral edema/lymphedema.

#### 3.1.4 Calcium Channel Blockers (Class IV)

Peripheral edema is a well-recognized effect of this class of antiarrhythmic drugs, which are used to control heart rate and slow conduction through the atrioventricular node. These medications also are effective vasodilator agents and their action on the lymphatic circulation is detailed in the next section.

### 3.2 Vasodilators

The calcium channel blockers (CCBs) have drawn the most interest as a major drug class of vasodilator medications linked to drug-related lymphedema. CCBs have dual anti-arrhythmic and direct vasodilator properties, and in addition to the control of cardiac arrhythmias, they are extensively used to treat several vascular diseases including hypertension and angina. A predominant side effect of CCBs is the formation of peripheral edema (Makani et al., 2011). One mechanism of edema formation may relate to the ability of CCBs to selectively dilate arterioles in the absence of venous dilation, ultimately leading to increased capillary pressure (Noll and Lüscher, 1998; Weir, 2003; Major et al., 2008). However, CCB-induced antagonism of L-type calcium channels in lymph muscle cells also attenuates the voltage-gated calcium influx required for rhythmic contractions; the reduced lymph flow may further contribute to the peripheral edema observed in patients subjected to CCB therapy. Many studies bear witness to the detrimental effect of CCBs on lymph vessel contractions and lymph flow. For example, the spontaneous contractions of isolated bovine mesenteric lymph vessels are inhibited by nifedipine and diltiazem, which represent CCBs from the distinctly different dihydropyridine and non-dihydropyridine classes of CCBs, respectively (Atchison and Johnston, 1997). Calculated intraluminal flow was correspondingly reduced by these two CCBs in the same studies. Similarly, nifedipine and diltiazem at nanomolar concentrations significantly reduced the amplitude and force of rhythmic contractions in rat mesenteric lymph vessels (Lee et al., 2014). Additional extensive studies have provided further evidence that CCBs inhibit lymphatic action potentials and contractions in mouse, guinea pig, rat, cow, and human isolated lymph vessels (Azuma et al., 1977; Atchison and Johnston, 1997; Imtiaz et al., 2007; Von Der Weid et al., 2008; Telinius et al., 2014c; Lee et al., 2014; To et al., 2020). Thus, calcium influx through L-type calcium channels is clearly a critical contributor to rhythmic contraction in collecting lymph vessels and clinically used CCBs potently inhibit these contractions and attenuate lymph flow.

In addition to L-type calcium channels, it should be acknowledged that lymph muscle cells appear to express transient (T-type) calcium channels, which may contribute to the upstroke of the action potential and pacemaker activity in some cell types. In this regard, pharmacological block of T-type calcium channels with nickel or mibefradil has been reported to reduce contraction frequency without affecting amplitude and force of contraction in isolated rat mesenteric lymph vessels (Lee et al., 2014). However, the low specificity of the blockers used in this study negate firm conclusions. Subsequent studies in isolated inguinal axillary and popliteal lymph vessels from genetically modified mice lacking the gene encoding T-type channels failed to support a prominent contribution of T-type calcium channels to contraction amplitude and frequency (To et al., 2020). Rather, these findings emphasized an indispensable role for L-type calcium channels in lymphatic contractile function.

Importantly, the essential role of L-type calcium channels in establishing the rhythmic contractions of lymph vessels that drive lymph flow extends to human lymph vessels. Clinically achievable concentrations of the dihydropyridine CCB, nifedipine, inhibits the spontaneous contractions of isolated human thoracic duct and mesenteric lymph vessels. The non-dihydropyridine calcium channel blocker, verapamil, also exerts inhibition but at concentrations in excess of those found clinically (Telinius et al., 2014c). The same authors then conducted a randomized, double-blinded, placebo-controlled cross-over to evaluate the role of lymphatic dysfunction in nifedipine-induced peripheral edema (Telinius et al., 2014c). No apparent evidence of lymphedema was observed in response to nifedipine administration in this study (Telinius et al., 2014c), but the small sample size (n = 6) was not sufficient to detect the 2–5% incidence of peripheral edema expected for the exposure period used in this study (Makani et al., 2011). A follow up randomized placebo-controlled trial in sixteen healthy, post-menopausal women investigated the effect of CCBs on lymphatic function (Mohanakumar et al., 2020). The women were treated with high-dose amlodipine for 3 months, lymphatic function was measured by near infrared imaging, and lower leg volume was determined by water displacement. No significant difference in lymphatic function (pumping pressure, contraction frequency, refill time, or velocity) was detected between the amlodipine-treated and placebo groups. However, seven out of the 16 study participants developed lower limb edema during treatment, and these seven subjects exhibited a lower baseline lymphatic pumping pressure than the subjects who did not develop edema (Mohanakumar et al., 2020). Based on these findings, the authors suggested that CCBs may not directly impair lymphatic function *in vivo*, but patients with impaired lymphatic function may be predisposed to CCB-associated edema/lymphedema.

In this regard, many conditions may compromise lymphatic function; for example, breast cancer treatments including surgery, radiation, and chemotherapy are associated with increased risk of lymphedema. Our group conducted a nested case-control study of adult female breast cancer patients to investigate the risk of lymphedema associated with CCB use in a population with predisposing factors (Stolarz et al., 2019a). A total of 717 cases and 1,681 matched controls were identified. Up to five controls were selected for each case and matched in the baseline period according to age (within 5 years), nest entry date (within 180 days), type of insurance, thiazide exposure, number of antihypertensive drug classes used and Charlson Comorbidity Index (CCI). In adjusted analysis, CCB administration was significantly associated with increased risk of lymphedema (OR = 1.320; 95% CI, 1.003–1.737). Subgroup analysis revealed no significant difference between dihydropyridine vs. non-dihydropyridine CCBs and lymphedema development. Thus, while acknowledging that our study had limitations (Stolarz et al., 2019a), the findings suggest that avoiding the use of CCBs in breast cancer patients may represent a viable strategy to prevent lymphedema. Findings from these studies and others support the concept that CCB-induced inhibition of L-type calcium channels in lymph muscle cells may disrupt lymphatic drainage and encourage accumulation of lymph fluid in interstitial spaces as a cause of lymphedema. These findings also raise the possibility that other medications that compromise calcium signaling or mechanisms of lymph muscle contraction may predispose patients to lymphedema and should be avoided in patients at risk as a component of medication management.

### 3.3 K_ATP_ Channel Openers

Pharmacological openers of adenosine triphosphate -sensitive potassium (K_ATP_) channels, which often are referred to as KCOs, are another class of medications strongly associated with peripheral edema (Sica, 2004; Nichols et al., 2013). The KCOs are primarily used to treat hypertension and to alleviate persistent hypoglycemia caused by excessive secretion of insulin from the pancreatic β cells (Nichols et al., 2013; Welters et al., 2015; Rubaiy, 2016). The molecular composition of K_ATP_ channels includes four pore-forming “Kir” subunits that confer potassium selectivity and are responsible for the property of inward rectification. Four ancillary sulfonylurea receptors (SUR) associate with the Kir subunits and bind intracellular regulators of the channel, and additionally contain the binding sites for KCOs (Ashcroft, 2005; Shi et al., 2012; Foster and Coetzee, 2015; Rubaiy, 2016). In vascular smooth muscle, the binding of KCOs to the SUR subunits results in K_ATP_ channel opening, potassium efflux, membrane hyperpolarization, closure of voltage-gated calcium channels, and vasodilation. This cascade of events leading to vasodilation accounts for the potent antihypertensive effect of some KCOs and their effectiveness in lowering blood pressure during hypertensive emergencies (Thirstrup and Nielsen-Kudsk, 1992). The binding of KCOs to the SUR subunit in pancreatic β cells similarly opens K_ATP_ channels to enact the same series of ionic events. In this cell type, however, the closure of voltage-gated calcium channels reduces the intracellular calcium concentration required for insulin secretion, thereby lowering circulating insulin and permitting blood glucose levels to rise toward normal levels (Ashcroft, 2005).

Two major side effects of KCOs that limit their value as drugs of choice for the treatment of hypertension and persistent hypoglycemia are peripheral edema and pericardial effusion, both diseases of disrupted fluid balance (Sica, 2004; Nichols et al., 2013). Similar to the CCBs and other vasodilator drugs, the development of peripheral edema during administration of KCOs may relate to vasodilator-induced increases in capillary pressure and subsequent movement of fluid from the capillaries into the interstitial space. However, growing evidence suggests that clinically achievable concentrations of KCOs also inhibit the rhythmic contractions of isolated lymph vessels and reduce *in vivo* lymph flow, raising the possibility that these off-target effects may contribute to the development of edema. For example, micromolar concentrations of cromakalim, a prototypical KCO, inhibit the rhythmic contractions of guinea pig mesenteric lymph vessels (Mathias and von der Weid, 2013). Similarly, KCOs abolish the rhythmic contractions of isolated human mesenteric lymph vessels (Telinius et al., 2014b). A recent report by our group detailed the effect on rat mesenteric lymph vessel contractions and *in vivo* lymph flow of commonly used clinical KCOs linked to severe peripheral edema (Garner et al., 2021). Minoxidil sulfate, an antihypertensive medication, inhibited the rhythmic contractions of isolated rat mesenteric lymph vessels. In the intact rat mesenteric lymphatic circulation, minoxidil sulfate reduced positive volumetric lymph flow at nanomolar concentrations corresponding to clinically achievable plasma concentrations in patients. Diazoxide, a drug of choice for the treatment of persistent hypoglycemia, also inhibited rhythmic contractions in isolated rat mesenteric lymph vessels (Garner et al., 2021). The higher concentration of diazoxide compared to minoxidil sulfate required to elicit this effect is consistent with diazoxide’s higher therapeutic plasma concentration range (Pohl and Thurston, 1973; The International Association of Forensic Toxicologists, 2013). Notably, other vasodilator drugs, such as hydralazine, that relax arterial muscle at least in part by opening K_ATP_ channels (Thirstrup and Nielsen-Kudsk, 1992; Bang et al., 1998), also are associated with edema formation (Maclean Ninewells Hospital and Vallance, 1986; Cohn et al., 2011), although the direct effects of hydralazine on lymphatic function have not been studied.

The recognition that disruption of lymph flow by KCOs may contribute to the development of edema raises the question of whether new drug design may be able to eliminate this off-target effect on the lymphatic circulation while retaining therapeutic efficacy. In this regard, the tissue-specific expression of different isoforms of K_ATP_ channel subunits may provide a mitigation strategy. Arterial K_ATP_ channels appear to be composed primarily of Kir6.1·SUR2B complexes (Shi et al., 2012; Foster and Coetzee, 2015; Rubaiy, 2016), whereas K_ATP_ channels in pancreatic β cells appear to represent Kir6.2·SUR1 assemblies (Ashcroft, 2005). The molecular composition of K_ATP_ channels in lymph muscle cells still is unclear but may be distinct from other cell types. Flow cytometry and patch-clamp studies from our group provide initial evidence that K_ATP_ channels in rat mesenteric lymph muscle cells may represent Kir6.1/6.2 co-assemblies potentially associated with SUR2B subunits (Garner et al., 2021), but further studies are needed to confirm these findings. Regardless, the growing awareness that many common cardiovascular drugs disrupt the lymphatic circulation implies that the design of new medications should consider this complication to minimize off-target effects.

## 4 Anti-Diabetic Agents

It is well known that diabetes and metabolic syndrome are associated with vascular dysfunction including vasoconstriction, inflammation, thrombosis, and atherosclerosis (Creager et al., 2003). In contrast, the prevalence of lymphatic dysfunction in diabetes is unknown although a correlation has been suggested (Mcintosh and Green, 2009). The presence of lymphatic dysfunction in animal models of metabolic syndrome is a recent discovery (Zawieja et al., 2012, 2015, 2016; Lee et al., 2020). For example, the pumping capability of isolated mesenteric lymph vessels was compromised and lymph vessels exhibited signs of remodeling in a rat model of metabolic syndrome (Zawieja et al., 2012). A follow-up study by the same authors showed reduced expression of endothelial nitric oxide synthase in the lymph vessels, which was associated with flow-mediated contraction in thoracic ducts isolated from rats with metabolic syndrome (Zawieja et al., 2015). Another study reported alterations in macrophages and increased K_ATP_ channel activity coupled to the impairment of lymphatic contractility in rats with metabolic syndrome (Zawieja et al., 2016). The latter finding raises the possibility that pharmacological block of K_ATP_ channels, which is the mechanism of action for the antidiabetic drug class known as sulfonylureas, may be useful to restore lymphatic function in metabolic syndrome. This topic will be discussed later in more detail (see Section 6.3). Finally a more recent study suggested that decreased activity of the Ca^2+^-ATPase pump, which helps to re-sequester calcium into the sarcoplasmic reticulum between action potentials, may be involved in the lymphatic contractile dysfunction observed in rats with metabolic syndrome (Lee et al., 2020). It is unclear to date whether these abnormalities of lymphatic function discovered by preclinical studies are features of the lymphatic circulation of diabetic patients and whether administration of common antidiabetic agents designed to control plasma glucose levels may have unintended effects on lymph flow. There is some evidence (as follows) that at least one class of antidiabetic agents, the thiazidolinediones may impair lymphatic function and contribute to the development of drug-induced secondary lymphedema.

### 4.1 Thiazodilinediones

Thiazidolinediones (TZDs) activate peroxisome proliferator activated receptor gamma (PPARγ), which is a transcriptional factor that upregulates genes responsible for glucose uptake (Vieira et al., 2019). TZDs are widely used for the treatment of type II diabetes, but one of their side effects is fluid retention and edema that occurs in a dose- dependent manner (DeFronzo, 1981; Yamanouchi, 2010; Yau et al., 2013). The incidence of edema is 5–10% in patients taking TZDs as a solo medication. However, administration of TZDs in combination with daily insulin increases edema incidence to 15–20% by an unknown interaction (Scheen, 2004). The mechanisms responsible for this drug-related edema are unclear, but a limited number of studies indicate that TZDs may directly affect lymphatic function. For example, the TZD rosiglitazone is reported to inhibit lymph-angiogenesis in a murine model of human gastric cancer (Chen et al., 2013). In this study, rosiglitazone dose-dependently reduced the lymph vessel density of implanted gastric tumors and decreased expression of VEGF-C and VEGFR-3, common lymphangiogenic markers (Chen et al., 2013). Another study evaluated the impact of rosiglitazone on lymphatic function by using technetium-labelled albumin to measure albumin retention and uptake of interstitial albumin by lymphatics in hindquarters of Zucker Diabetic Fatty (ZDF) rats (Cosson et al., 2009). In previous studies the ratio of low and high frequency peaks (LF/HF ratio) correlated with the half-life of the technetium-labelled albumin disappearance during lymphoscintigraphy (Cohen-Boulakia et al., 2000; Pecking et al., 2008); therefore, LF/HF ratio was used as a surrogate index of lymphatic uptake of albumin. The hind limbs of these diabetic animals exhibited albumin retention and a high LF/HF ratio (which inversely correlates with lymph uptake) as evidence of compromised drainage of lymph fluid. Treatment with rosiglitazone resolved the albumin retention but did not restore lymph uptake to control levels. One interpretation of these data is that rosiglitazone lowers albumin retention by decreasing capillary filtration but does not modify lymphatic uptake. Collectively, these initial studies provide little firm insight into mechanism(s) by which rosiglitazone causes edema in clinical practice, but they serve to emphasize that medications associated with the development of edema may act at multiple sites to disrupt local fluid dynamics including lymph vessel density, capillary filtration, lymph uptake and other processes that maintain the delicate fluid balance in the interstitial space.

## 5 Chemotherapeutic Agents

Cancer-related lymphedema is one of the most common types of secondary lymphedema. This type of lymphedema often is attributed to surgical interventions that physically damage lymph vessels to disrupt lymph flow. However, recent studies have provided evidence that cancer-related lymphedema is a multi-hit process, with each additional cancer treatment modality, like radiation and chemotherapy, increasing the risk of lymphedema (Nguyen et al., 2017). Many prospective and retrospective observational trials have studied the risk of lymphedema associated with chemotherapy use; however, only a handful of studies have investigated the direct effects of chemotherapy agents on lymphatic contractile function and lymph flow. These more mechanistic studies play a vital role in implicating specific chemotherapeutic agents in the pathogenesis of lymphedema, which is difficult to establish in patient populations due to the confounding nature of epidemiological data derived from complex clinical settings. For example, patients are often treated with a cocktail of different chemotherapeutic drugs, making it difficult to isolate the contribution of individual agents to the development of lymphedema. For example, in breast cancer, which carries a 30% incidence of arm lymphedema, patients are commonly treated with a combination drug regimen containing an anthracycline, a taxane, and an alkylating chemotherapeutic agent. These classes of chemotherapeutic drugs are widely used in combination to treat many different types of cancers, making it difficult to attribute increased risk of cancer-related lymphedema to a single medication. Still, considerable scientific and clinical evidence points to chemotherapeutic agents as contributors to the development of lymphedema in cancer patients subjected to their beneficial and off-target effects.

### 5.1 Anthracyclines

There is considerable evidence that anthracycline drugs, particularly doxorubicin, contribute significantly to lymphedema risk in cancer patients (Norman et al., 2010; Ahmed et al., 2011; Bevilacqua et al., 2012; Nguyen et al., 2017). Doxorubicin is often referred to as the “red devil” not only because it forms a red solution when reconstituted in solution before administration, but also because it causes a myriad of serious adverse effects including cardiotoxicity, myelosuppression, and lymphedema. Doxorubicin is used to treat a variety of malignancies including but not limited to Hodgkin’s disease, acute lymphoblastic leukemia, breast cancer, genitourinary cancers, and gynecological cancers (Young et al., 1981; Minotti et al., 2004; Cortés-Funes and Coronado, 2007; Bedford, 2012; Acheson, 2015). Although administration of anthracyclines may be associated with an increased risk of lymphedema in all of these malignancies, most studies have focused on the contribution of anthracyclines to breast-cancer related lymphedema, where doxorubicin is the anthracycline of choice. For example, in a 2010 prospective cohort study that followed 631 breast cancer patients over 5 years, addition of anthracycline chemotherapy to surgical interventions increased the risk of lymphedema by 4 to 5-fold regardless of irradiation status (Norman et al., 2010). In fact, the addition of anthracycline chemotherapy to the treatment of patients subjected to sentinel lymph node biopsy without radiation increased the risk of lymphedema to levels comparable to patients subjected to the more radical surgical procedure of axillary lymph node dissection (Norman et al., 2010). In a more recent larger cohort study that assessed risk factors for lymphedema in 1794 breast cancer patients followed for a median of 10 years, treatment with anthracyclines was associated with a 13.5% incidence of lymphedema by 5 years (Nguyen et al., 2017). Moreover addition of anthracyclines like doxorubicin to the treatment regimen increased the 5 years incidence of lymphedema by more than 2-fold in patients who underwent axillary lymph node dissection and increased lymphedema by more than 3-fold in patients who additionally received radiotherapy (Nguyen et al., 2017).

Doxorubicin’s therapeutic and cytotoxic action is mediated by DNA intercalation and generation of oxidative stress at the tumor site. The planar anthracycline structure of doxorubicin permits specific binding to base pairs of the DNA double helix causing steric hindrance and preventing DNA cleavage by topoisomerase-II. Doxorubicin also chelates iron to yield hydroxyl free radicals that mediate damage to DNA and other cellular structures (Young et al., 1981; Minotti et al., 2004; Thorn et al., 2011). It was assumed until recently that the cytotoxic actions of doxorubicin also damaged the lymphatic circulation and accounted for the higher incidence of lymphedema in patients administered this chemotherapeutic drug. However, our group recently reported that doxorubicin directly impairs the rhythmic contractions of isolated lymph vessels and compromises *in vivo* lymph flow as an off-target effect (Stolarz et al., 2019; Van et al., 2021). Notably, earlier studies provided evidence that doxorubicin directly alters calcium signaling in skeletal muscle cells and cardiac myocytes by tonically activating ryanodine receptors to mediate release of intracellular calcium stores (Zorzato et al., 1985, 1986; Abramson et al., 1988; Pessah et al., 1990; Saeki et al., 2002). Building on these findings, we demonstrated that clinically achievable concentrations of doxorubicin acutely inhibit rhythmic contractions in isolated rat mesenteric collecting lymph vessels and reduce flow in the *in situ* mesenteric lymphatic circulation (Stolarz et al., 2019). Additional studies revealed that doxorubicin tonically increased cytosolic free calcium in lymph muscle cells as a mechanism to disrupt the cyclic rises in intracellular calcium required for rhythmic contractions and normal lymph flow. Importantly, the tonic rise in cytosolic free calcium was prevented by pharmacological antagonists of ryanodine receptors but not by blocking other calcium-mobilizing channels (Stolarz et al., 2019). Interestingly, this ability to disrupt Ca^2+^ signaling also is a feature of other anthracycline medications (Olson et al., 2000; Shadle et al., 2000; Hanna et al., 2011), raising the possibility of a shared effect of anthracyclines on lymphatic contractile function that may to contribute the development of secondary lymphedema. Earlier studies suggest that the disruption of lymphatic contractions and the resulting lymphostasis damages the lymphatic endothelium and fragile lymphatic valve leaflets, setting the stage for long-term compromise of lymph vessel contractile function (Zawieja et al., 1991; Ji and Kato, 2001; Gashev, 2002; Nipper and Dixon, 2011; Choi et al., 2012).

A follow-up study by our group investigated the ability of dantrolene to prevent the detrimental effects of doxorubicin on the lymphatic circulation (Van et al., 2021). Dantrolene is a ryanodine receptor type 1 (RYR1) antagonist used clinically to mitigate malignant hyperthermia (Rosenberg et al., 2015; Ratto and Joyner, 2021). However, we considered that this drug could be repurposed as a prophylactic to prevent anthracycline-associated lymphedema. Initial studies confirmed that the molecular target of dantrolene, the RYR1 protein, was expressed by single lymph muscle cells freshly isolated from rat mesenteric lymph vessels. Subsequently dantrolene was observed to prevent the pathogenic rise in intracellular calcium elicited by doxorubicin in isolated lymph vessels. Finally, topical superfusion of the intact mesenteric lymphatic circulation with dantrolene prevented the interruption of lymph flow caused by systemic infusion of doxorubicin (Van et al., 2021). Future longitudinal studies are necessary to evaluate dantrolene as an anti-lymphedema medication, but these initial findings raise the possibility that dantrolene co-administered during chemotherapy may mitigate the detrimental effects of doxorubicin on the lymphatic circulation to preserve lymph flow.

### 5.2 Taxanes

Taxanes are a common class of chemotherapy drugs that are well known to cause fluid retention (Béhar et al., 1997; Ho and Mackey, 2014). This drug class, including paclitaxel and docetaxel, is widely used to treat many cancers including breast, lung, head and neck, and prostate cancer (Ojima et al., 2016). Several epidemiological studies have linked taxane chemotherapy to an increased incidence of breast cancer related lymphedema (Lee MJ. et al., 2014; Nguyen et al., 2017; Zhang et al., 2019). For example, in a longitudinal study of 63 breast cancer patients who received axillary surgery and a chemotherapy regimen containing anthracyclines and taxanes, 25–30% of patients developed lymphedema within the first 6 months after completing taxane therapy (Lee MJ. et al., 2014). In a larger cohort study that assessed risk factors for lymphedema in 1794 breast cancer patients, the overall 5-year incidence of lymphedema in patients treated with taxane chemotherapy was approximately 30% (Nguyen et al., 2017). Moreover, addition of taxanes to any treatment regimen resulted in a 33–40% incidence of lymphedema at 5 years (Nguyen et al., 2017).

Taxanes, exert their cytotoxic actions by stabilizing microtubule formation thereby inhibiting mitosis and resulting in apoptosis (Ojima et al., 2016). Taxanes also inhibit tumor angiogenesis, an action that may be partially attributed to their ability to inhibit endothelial cell migration and proliferation (Bocci et al., 2013). These inhibitory actions on endothelial cells may also partially explain the association of taxane drugs with lymphedema, although the literature fails to clarify the mechanism by which this class of drugs may injure the lymphatic circulation. In this regard, a recent study indicates that the common taxane medication, paclitaxel, induces autophagy in human dermal lymphatic endothelial cells (HDLECs) and disrupts gap junctions correlating to loss of the gap junction protein, VE-cadherin. Based on these findings, the authors suggest that paclitaxel disrupts the endothelial barrier dysfunction to increase the permeability of lymph vessels (Zamora et al., 2019). The same authors also reported that paclitaxel modestly attenuates tumor lymph-angiogenesis in a tumor-bearing mouse model, apparently confirming significant drug effects on the lymphatic endothelium albeit in a specific pathological setting (Zamora et al., 2019). Finally, a subsequent report that docetaxel, another commonly used taxane medication, attenuates proliferation and inhibits migration and tubule formation of HDLECs in primary culture provides additional evidence that taxanes may impair endothelial function and limit lymph-angiogenesis (Wong et al., 2020). Indeed, the authors also noted a decreased expression of the endothelium-specific marker LYVE-1 in cultured HDLECs exposed to docetaxel, although expression of Prox-1, another lymphatic endothelium-specific marker was unchanged (Wong et al., 2020). These initial *in vitro* findings linking taxanes to injury of the lymphatic endothelium have set the stage for proof-of-principle studies to examine the impact of systemic taxane administration on endothelial integrity, lymphatic function and *in vivo* lymph flow.

### 5.3 Corticosteroids Co-Administered With Chemotherapy

Administration of corticosteroids during chemotherapy regimens, particularly longer courses of corticosteroid treatment, also is correlated with an increased incidence of cancer-related lymphedema. However, the diverse actions of this class of medications include activation of mineralocorticoid receptors to promote fluid retention (Schimmer and Funder, 2017), which *per se* could disrupt fluid balance and contribute to lymphedema independently of other effects on the blood and lymphatic circulations. Common clinical protocols utilize pre-medication with corticosteroids prior to chemotherapy as a prophylactic strategy to deter infusion reactions (Boulanger et al., 2014). A correlation between corticosteroid use and cancer-related lymphedema has only been anecdotally reported, and no dedicated studies have directly assessed the role of corticosteroids in cancer related lymphedema. Interestingly, other anti-inflammatory and immunosuppressive agents are being investigated as *potential treatments* for lymphedema (as described in the next section), suggesting that properties unique to the corticosteroids may account for their apparent involvement in the development of lymphedema.

## 6 Potential Therapies for Lymphedema

There are no FDA-approved pharmacological agents to treat or prevent lymphedema. However, several classes of medications are administered to attempt to alleviate lymphedema in patients with co-morbid conditions. Other compounds are in the pipeline with the intent of introducing new drugs or repurposing existing drugs to target specific causative mechanisms of lymphedema. The following section will briefly review some of these therapeutic approaches. Table 2 summarizes the potential therapies for lymphedema included in this review.

**TABLE 2:** Stage of development for potential therapeutic agents for lymphedema.

Therapeutic agents	Potential mechanism of action in lymphedema	Phase of development	References
BayK8644	Activate L-type Ca^2+^ channels	Discovery and development	Zawieja et al. (2018)
Dantrolene	Block ryanodine receptor 1	Discovery and development	Stolarz et al. (2019)
			Van et al. (2021)
Diuretics	Reduce intravascular fluid volume	Limited clinical use due to rebound edema	Harris et al. (2001)
Glibenclamide	Block K_ATP_ channels	Discovery and development	Zawieja et al. (2016)
			Davis et al. (2020)
Ketoprofen	Anti-inflammatory; possible inhibition of 5-lipoxygenase-LTB4 pathway	Phase IV, limited clinical use	Muacevic and Adler (2019)
			Rockson et al. (2018)
			Nakamura et al. (2009)
Recombinant human hyaluronidase	Breaks down the polymeric structure of hyaluron	Phase II, terminated	Makani et al. (2011)
Tacrolimus	Prevent T cell infiltration and inflammation in the tissue region	Phase II	Gardenier et al. (2017)

### 6.1 Diuretics

Diuretics are the most common pharmacological approach to treat conditions involving edema or fluid retention. However, diuretics appear to be of limited clinical use in the treatment of lymphedema, except in patients with certain co-morbid conditions including heart failure and cirrhosis (Harris et al., 2001). The limited clinical utility of diuretics in the treatment of lymphedema may relate in part to the high osmotic pressure of fluid in the interstitial space distal to impaired lymphatic drainage, which is caused by protein accumulation in the extracellular space. The elevated osmotic pressure may facilitate rebound fluid accumulation after diuretic administration to maintain and perpetuate the development of lymphedema (Harris et al., 2001). Meanwhile the systemic effects of diuretics may lead to electrolyte imbalances, dehydration, and hypotension in already compromised patients (Harris et al., 2001).

### 6.2 Hyaluronidase

A Phase I/II clinical trial initiated in 2013 was designed to evaluate the safety and efficacy of recombinant human hyaluronidase injection (rHUPH20) for the treatment of cancer-related lymphedema. Hyaluronic acid or hyaluron is a polysaccharide polymer and a chief component of the extracellular matrix. It increases osmotic pressure, thereby elevating the resistance to flow of extracellular fluid to establish a positive fluid balance (Aukland and Reed, 1993; Laurent et al., 1996). The actions of hyaluron are normally counteracted by the enzyme hyaluronidase, which actively breaks down the polymeric structure of hyaluron to afford increased fluid flow (Aukland and Reed, 1993; Laurent et al., 1996). Interestingly, hyaluron has been shown to accumulate during edema associated with transplant rejection (Tufveson et al., 1992). Accordingly, a Phase I/II clinical trial was conducted to evaluate the benefit of administering recombinant human hyaluronidase (rHUPH20) to transplant patients suffering from this form of edema. rHPH20 was administered by subcutaneous injection on days 1, 3, 5, and 7 during Phase I, followed by daily subcutaneous injections for 21 consecutive days during Phase II. The study was terminated due to low accrual with only three participants recruited during the course of the study (Pegram, Stanford University, 2017). However, conjecture suggests that while hyaluronidase may have reduced limb circumference, its subcutaneous injection could be regarded to increase the risk of infection, a finding noted anecdotally in other studies (Cemal et al., 2011; Inghammar et al., 2014; Kilbreath et al., 2016) and reflected in consensus recommendations (Harris et al., 2001; The diagnosis and treatment of peripheral lymphedema, 2013 Consensus document of the international society of lymphology, 2013). However, the concept of manipulating osmotic pressure to restore interstitial fluid balance and mitigate lymphedema may have merit in other contexts.

### 6.3 Ion Channel Modulating Agents

This chapter has emphasized that calcium influx mediated by voltage-sensitive L-type calcium channels in lymph muscle cells mediates the lymphatic contractions that facilitate lymph flow. Accordingly, investigations have evaluated the benefit of using calcium channel agonists to enhance lymph vessel contractions and increase lymph flow. BayK8644, a dihydropyridine analog that opens L-type calcium channels to promote calcium influx has garnered particular interest. Preclinical studies demonstrate that BayK8644 prolongs action potential durations in lymph muscle cells, increases amplitude and duration of calcium transients, and shortens the time to peak calcium concentration in isolated murine lymph vessels (Zawieja et al., 2018). Although these actions of BayK8644 may enhance lymphatic contractions, the systemic utility of this drug may be limited due to the expression of L-type calcium channels in cardiac muscle and vascular smooth muscle, where their pharmacological activation may cause cardiac arrhythmias, vasoconstriction, hypertension, and other unwanted events (Seifen and Kennedy, 1986; Gao et al., 2011; Borysova et al., 2018; Koleske et al., 2018). Additionally, it may be difficult to titrate the concentration of BayK8644 to avoid “hyper-activation” of L-type calcium channels, which could result in tonic constriction of lymph vessels and reduced lymphatic drainage.

As mentioned earlier (see Sections 2, 4), K_ATP_ channel blockers may be useful to restore lymphatic function in diabetes and other conditions characterized by the excessive opening of K_ATP_ channels (Zawieja et al., 2016; Davis et al., 2020). One specific antagonist of K_ATP_ channels is glibenclamide, which is a common antidiabetic agent. In a study using rats with metabolic syndrome, glibenclamide partially restored the frequency of lymphatic contractions and fractional pump flow in isolated mesenteric lymph vessels (Zawieja et al., 2016). Similarly, glibenclamide exerts an anti-lymphedema action in Cantu syndrome, which is a disease in which K_ATP_ channels exhibit gain-of-function (i.e., high open-state probability) associated with a high incidence of lymphedema (Nichols et al., 2013). In a genetic mouse model with this gain-of-function mutation in the K_ATP_ channel, glibenclamide partially restored normal resting membrane potential in lymph muscle cells and improved rhythmic contractions in isolated popliteal lymph vessels (Davis et al., 2020). In contrast, glibenclamide does not appear to affect contractions in lymph vessels isolated from control rats and humans (Mizuno et al., 1999; Mathias and von der Weid, 2013; Telinius et al., 2014b). More studies are needed to characterize the therapeutic potential of glibenclamide and other K_ATP_ channel blockers to preserve lymphatic function in patients with metabolic diseases, Cantu syndrome and other diseases in which K_ATP_ channel activity may be abnormally high.

Dantrolene is another calcium ion channel modulator that has been investigated preclinically as a potential anti-lymphedema agent to prevent the inhibitory effects of doxorubicin on lymphatic contractile function (see Section 5). Dantrolene is a pharmacological antagonist of the ryanodine receptor 1 (RYR1) channel, which is approved by the FDA to treat malignant hyperthermia and muscle spasm (Rosenberg et al., 2015; Ratto and Joyner, 2021). Preclinical studies in isolated rat mesenteric lymph vessels suggest that dantrolene-induced block of RYR1 fails to significantly alter spontaneous rhythmic contractions under resting conditions (Jo et al., 2019; Stolarz et al., 2019; Van et al., 2021), but protects against doxorubicin-induced activation of RYR1 and the induction of “calcium leak” that disrupts cyclic calcium signaling and rhythmic contractions in lymph muscle cells. The additional finding that dantrolene co-administered with doxorubicin preserves lymph flow, suggest that dantrolene or other ryanodine receptor blockers may be ideal candidates to prevent the lymphedema associated with anthracycline chemotherapy (Stolarz et al., 2019; Van et al., 2021).

### 6.4 Anti-inflammatory and Immunosuppressive Agents

It is well known that lymphedema is associated with chronic inflammation and a regional immune response. Accordingly, anti-inflammatory and immunosuppressive agents have been considered as potential therapies to treat lymphatic vascular insufficiency and lymphedema (Forte et al., 2019). Ketoprofen, a non-steroid anti-inflammatory drug (NSAID) has demonstrated a remarkable ability to promote lymphangiogenesis, improve lymphatic function and reduce lymphedema in animal models and clinical trials (Nakamura et al., 2009; Rockson et al., 2018; Forte et al., 2019). Accordingly, there are additional clinical trials in progress to evaluate the effect of ketoprofen on lymphedema. Interestingly, these beneficial effects do not appear to extend to other NSAIDs. For example, the modified soluble TNFa receptor, pegsunercept, failed to improve experimental lymphedema in mice (Rockson et al., 2018). Moreover, the clinical usage of ketoprofen to treat lymphedema is limited by its renal, hepatic and central nervous system toxicities (Tian et al., 2017). Identification of the precise downstream mechanism by which ketoprofen ameliorates lymphedema may enable the design of improved anti-lymphedema agents targeting specific pathways implicated in the underlying disease process. In this regard, a promising recent report indicates that the anti-lymphedema effect of ketoprofen may be attributed to inhibition of the 5-lipoxygenase - leukotriene B4 (LTB4) pathway, which may represent a new therapeutic target (Tian et al., 2017).

Another immune suppressive agent explored as an anti-lymphedema medication is the T cell inhibitor tacrolimus (Gardenier et al., 2017). Topical application of tacrolimus is reported to prevent T cell infiltration and inflammation in the adjacent tissue and promote lymphangiogenesis (Gardenier et al., 2017). The precise molecular mechanism by which tacrolimus contributes to lymphangiogenesis remains to be identified, and further investigations may uncover unique molecular targets for future drug development. Additionally, new clinical trials are being conducted to investigate the effect of tacrolimus in breast cancer related lymphedema. Collectively, these early studies provide hope that novel medications will be identified in the near future to effectively prevent the development and lasting impact of lymphedema.

## 7 Discussion/Conclusion

Many factors cause disruption of lymph transport including genetic malformations/mutations, physical injury to lymph vessels, surgical removal of lymph vessels, and administration of certain medications. There is growing recognition that many pharmacological agents act on ion channels and other protein structures in lymph muscle cells to disrupt the cyclic contraction and relaxation of collecting lymph vessels, thereby compromising lymph flow and contributing to the development of lymphedema. Interpretation of findings from clinical studies exploring factors contributing to the development of drug-related lymphedema is confounded by the presence of comorbid conditions that also may disrupt fluid balance. However, the insight gained from these studies combined with mechanistic exploration of factors and medications that modify rhythmic contractions in isolated lymph vessels and alter lymph flow in the intact lymphatic circulation will lead to an appreciation of the pathogenesis of lymphedema and new strategies to treat it.
